# Changes in Lower Limb Biomechanics Across Various Stages of Maturation and Implications for ACL Injury Risk in Female Athletes: a Systematic Review

**DOI:** 10.1007/s40279-024-02022-3

**Published:** 2024-04-26

**Authors:** Akhilesh Kumar Ramachandran, Jason S. Pedley, Sylvia Moeskops, Jon L. Oliver, Gregory D. Myer, Rhodri S. Lloyd

**Affiliations:** 1 Youth Physical Development Centre, Cardiff School of Sport and Health Sciences, Cardiff Metropolitan University, Cyncoed Campus, Cyncoed Road, Cardiff, CF23 6XD UK; 2 Sport Performance Research Institute, New Zealand (SPRINZ), AUT University, Auckland, New Zealand; 3 Centre for Sport Science and Human Performance, Waikato Institute of Technology, Hamilton, New Zealand; 4 Emory Sports Performance and Research Center (SPARC), Flowery Branch, GA USA; 5 Emory Sports Medicine Center, Atlanta, GA USA; 6 Department of Orthopaedics, Emory University School of Medicine, Atlanta, GA USA; 7 The Micheli Center for Sports Injury Prevention, Waltham, MA USA

## Abstract

**Background:**

Female athletes are four to six times more likely to sustain an anterior cruciate ligament (ACL) injury than male athletes. Jump-landing biomechanics are influenced by maturation, with post-pubertal female athletes at a heightened risk of ACL injuries.

**Objective:**

The aim of our systematic review was to identify and summarise the current evidence regarding the changes in kinematic and kinetic risk factors associated with ACL injuries during jump-landing tasks in female athletes at various stages of maturity.

**Methods:**

A systematic search was conducted in PubMed, CINAHL, Web of Science, SPORTDiscus, EMBASE and Scopus. Articles were included if they: (1) conducted the research on uninjured female athletes with no restriction on playing level/experience; (2) provided information regarding the stage of the maturity and the scale used for estimating the maturity status of the participants; and (3) reported a biomechanical risk factor associated with ACL injuries during jump-landing tasks across at least two different maturity groups (e.g. pre-pubertal vs post-pubertal).

**Results:**

Sixteen articles involving 2323 female athletes were included in our review. A total of 12 kinematic and 8 kinetic variables were identified across these studies. Of the 12 kinematic variables reported in our review, we found strong evidence for higher peak knee abduction angle in post-pubertal female individuals compared with pre-pubertal girls (*p* < 0.05). With regard to the 8 kinetic variables, we found strong evidence for lower relative peak vertical ground reaction force, higher external knee abduction moment and internal rotation moment in post-pubertal compared with pre-pubertal athletes. The strength of evidence for the remaining kinematic and kinetic variables ranged from conflicting to moderate and, in some instances, could not be determined.

**Conclusions:**

Our study provides an overview of the changes in biomechanical risk factors in female athletes during jump-landing tasks at various stages of maturity. We found moderate-to-limited evidence for most kinematic and kinetic variables, highlighting the need for further research.

**Supplementary Information:**

The online version contains supplementary material available at 10.1007/s40279-024-02022-3.

## Key Points

**Table:** 

There was strong evidence for post-pubertal female athletes to have higher peak knee abduction angle compared with pre-pubertal athletes.
There was strong evidence for post-pubertal athletes to have higher peak external knee abduction moment and internal rotation moment compared with pre-pubertal athletes.
There was strong evidence for pre-pubertal athletes to have higher relative vertical ground reaction force compared with post-pubertal athletes.
It is recommended that future research should explore the changes in jump-landing biomechanics across hip and ankle joints in addition to the knee joint across various maturity levels in female athletes.

## Introduction

Anterior cruciate ligament (ACL) injuries have been traditionally considered to be of limited concern in skeletally immature athletes based on the relative low incidence [1]. However, more recent research findings have shown trends of an increased incidence in ACL injury rates in this population. For example, in Australia, the rate of ACL injuries in children aged between 5 and 14 years increased by 148% between 2005 and 2015 [2]. In Italy, the overall percentage of ACL reconstruction surgeries in children aged younger than 15 years has increased from 0.13 to 0.95% between 2001 and 2015, with 97.3% of these surgeries being performed on children aged 10–14 years [3]. Apart from the financial burden associated with ACL injuries, there is an increased risk of longer term health issues. Athletes with prior knee injuries have been reported to have negative health-related outcomes including knee-related pain, poor quality of life and a higher body mass index [4]. Further, some youth who have an ACL injury experience an early onset of osteoarthritis within 15 years of the injury [5–7]. In young athletes, the risk of secondary ACL injury has been reported to be as high as 25–35% [8–10] within 2–5 years of the first injury, underlining the importance of early risk mitigation.

Anterior cruciate ligament injuries commonly occur during the deceleration phase of dynamic movements such as single-leg (SL) or double-leg jump-landing [11–13]. The majority of ACL injuries during these movements occur in non-contact scenarios [14], and are usually a consequence of poor multiplanar biomechanics [14]. Reduced knee flexion angle, greater knee abduction angle and moments and peak vertical ground reaction force (vGRF) during jump-landing tasks are biomechanical factors associated with an increased risk of sustaining a non-contact ACL injury [15]. A prospective biomechanical and epidemiological investigation showed that adolescent female athletes that sustained ACL injuries demonstrated a knee abduction angle that was 8° greater at initial contact (IC) during a jump-landing task as compared with their uninjured peers [15]. The female athletes who went on to sustain a ACL injury also landed with a 2.5 times higher peak knee abduction moment and 20% greater ground reaction forces prior to injury compared with uninjured teammates [15]. Despite research being conducted in this domain for over two decades, the findings are still inconclusive as the aforementioned variables have only shown associations with ACL injury and as such cannot be considered as an ACL injury risk factor, [16, 17]. Recent literature highlighted that more high-quality research studies (i.e. prospective studies, level 1 evidence) related to biomechanical risk factors for non-contact ACL injuries need to be conducted as the majority of existing evidence comes from lower quality studies (i.e. retrospective, level 2 evidence) or associative study designs (i.e. level 3) [16]. Further, the biomechanics and the forces acting on the knee joint can be altered by the motion occurring across the hip and ankle joint [18]; however, few studies have reported kinematic and kinetic variables across all three joints [16, 17, 19]. Therefore, further exploring the biomechanical risk factors during jump-landing tasks across various planes of motion at the hip, knee and ankle joints will help to better understand the contributing risk factors to ACL injuries.

The literature suggests that changes in anatomy [20], joint biomechanics [21], hormones [22, 23] and neuromuscular control [24, 25] during the adolescent growth spurt could potentially influence the rates of ACL injuries. This is a period when the long bones (tibia and femur) grow at a rapid rate resulting in increased stature [26]. The pubertal growth spurt leads to longer levers, which translate into greater potential for increased torques on the knee joint [21]. Further, the increased limb length leads to a higher centre of mass, which makes muscular control of body position more challenging [27]. In addition, the ability to balance and dampen forces during high-velocity movements (such as jump-landing tasks) becomes more difficult and therefore injury risk is heightened [21]. As female individuals transition though the various stages of adolescence and reach the latter stages of maturity, they typically land with less knee flexion and higher ground reaction forces and external knee moments, thus putting them at a higher risk of sustaining an ACL injury [28]. The absence of sufficient neuromuscular control to stabilise the knee while performing activities involving large forces and torques might partly explain the increased incidence of ACL injuries in post-pubertal female individuals compared with prepubertal and pubertal female athletes [15, 25]. Hence, identifying the magnitude of variation in biomechanical risk factors during jump-landing tasks relative to maturational development might help to understand if these risk factors are present during the childhood years or are exacerbated as a result of growth and maturation in female athletes.

To date, the literature is limited to isolated investigations without any syntheses that provide comprehensive conclusions related to the influences of maturation on multi-joint landing mechanisms in female athletes. Therefore, the primary objective of our systematic review was to identify and summarise the current scientific evidence regarding the changes in kinetic and kinematic risk factors associated with ACL injuries during jump-landing tasks in female athletes at various stages of maturity.

## Methods

This review was conducted in accordance with Preferred Reporting Items for Systematic Review and Meta-analyses (PRISMA) [29]. A review protocol was not pre-registered for this review. This systematic review focused on providing a comprehensive summary regarding the changes in biomechanical variables across various maturity groups during jump-landing tasks in female athletes.

### Eligibility Criteria

The inclusion criteria were based on the population, intervention, comparator, outcome and study design (PICOS) concept as follows, whereby studies needed to: (1) conduct the research on uninjured female athletes with no restriction on playing level/experience; (2) provide information regarding the stage of maturity and the scale used for estimating the maturity status of the participants; and (3) report a kinematic (e.g. joint angles at various instances such as IC, peak values, range of motion/displacement) and/or kinetic variables (e.g. absolute or relative forces, absolute or relative moments) during jump-landing tasks across at least two different maturity groups (e.g. prepubertal vs post-pubertal; pubertal vs post-pubertal).

The exclusion criteria for the review were as follows: (1) studies that did not report the maturity status of the participants; (2) studies that did not include female athletes; (3) studies that reported biomechanical variables during non-jump-landing tasks, such as side-stepping and cutting; (4) studies in which no biomechanical variables were reported during jump-landing tasks; (5) studies that reported results based on simulation models; (6) book chapters, reviews, systematic reviews and meta-analyses, conference proceedings, poster presentations, conference abstracts, reviews, clinical commentaries, theses and dissertations; and (7) articles not published in English.

### Information Sources and Search Strategy

A systematic literature search was conducted across the following scientific databases to identify original research articles published from inception to July 2022 and then updated in May 2023: PubMed, CINAHL, Web of Science, SPORTDiscus, EMBASE and Scopus. The Boolean operator ‘AND’ and ‘OR’ were used to combine the various search terms. The complete search strategy across the different databases has been provided in Appendix S1 of the Electronic Supplementary Material (ESM). The reference lists of the included studies were also screened by one author (AKR) to identify any additional studies that were relevant for this review.

### Selection Process

One author (AKR) carried out the search across all the relevant databases. Potential titles and abstracts were imported into Endnote (Version 20; Clarivate, Philadelphia, PA, USA) and the duplicate articles were removed. The articles were then screened according to the inclusion/exclusion criteria. A three-stage process was followed to identify the relevant articles. First, articles were included in the first stage if they had investigated biomechanics related to ACL injuries, and jump-landing tasks. Second, the abstract of each study was then screened and the studies that did not report findings on female participants were excluded. The third and final stage involved reviewing the full text of all relevant studies that satisfied the eligibility criteria to scrutinise their suitability for final inclusion. Two authors (AKR and RSL) independently performed all these tasks. All potential discrepancies regarding the inclusion/exclusion of studies were discussed between the two authors and resolved. Another member of the authorship team (JLO) was identified to consult in the event that any discrepancies could not be resolved.

### Data Extraction and Reduction

The following data were extracted from the included articles: (1) author name, year of publication; (2) age, stature, mass and maturity status of the participants; (3) sporting activity and level; (4) various jump-landing tests that were used; (5) mode of data collection; (6) measurement units; (7) kinematic and kinetic data that were analysed; and (8) mean and standard deviation for each of the biomechanical risk factors across the various maturity groups.

When the data were reported in a graphical format, WebPlotDigitiser (https://automeris.io/WebPlotDigitizer/) was used to derive the numerical data. This procedure has proven to be valid (*r* = 0.99, *p* < 0.001) in previous studies [30]. In cases where studies had reported 95% confidence intervals, the recommended formula presented in the Cochrane handbook [31] was used for obtaining the standard deviation values:

$${\text{Standard deviation }} = {\text{ sqrt }}\left( N \right) \, \times \, \left( {\left( {{95}\% {\text{ CI upper limit }} - { 95}\% {\text{ CI lower limit}}} \right)} \right)/{3}.{92}$$

In cases where the standard error was reported, the following formula was used to convert it to a standard deviation, where *N* is the sample of the respective group (pre-pubertal, pubertal or post-pubertal):

$${\text{Standard deviation }} = {\text{ standard error }} \times {\text{ sqrt }}\left( N \right)$$

With regard to the kinetic variables, absolute joint moments were normalised using various techniques such as dividing moments by body weight [BW] (Nm/kg), BW times height (dimensionless) or BW times leg length (Nm/m), with the methods being inconsistent among researchers [32]. When a study reported values using different normalising techniques, moments normalised to BW (Nm/kg) were included in our review as this is the most commonly used technique used to report kinetic findings in biomechanical research [32].

### Quality Assessment of Included Studies

Two authors (AKR and RSL) independently carried out the quality assessment of the included studies. The Downs and Black checklist [33] was used for the methodological quality assessment of the included studies (ESM). The original checklist consists of 27 items that address methodological components, including external validity, internal validity (bias and confounding variables) and power. The quality index of the checklist has been reported to have a high criterion validity (*r* = 0.90), high internal consistency (KR-20 = 0.89), test–retest (*r* = 0.88) and inter-rater (*r* = 0.75) reliability. However, we used the modified version of this checklist consisting of 17 items [34], which has been used previously in research because of the subjectivity in interpretation of the original questions. The question for power was modified from a scale of 0–5 to a binary scale, in which all questions were given a score of 0 (no) or 1 (yes). However, question 20 was given a score of 0 (no), 1 (partial) or 2 (yes); a score of 2 was given when the study had reported the accuracy and described the methods clearly, while 1 point was given when only the methods were described. These modifications resulted in a total critical appraisal score of 18 points for the assessment of the included studies. The points obtained using the Downs and Black rating scale were converted to a percentage score, with studies classified as high (≥ 71%), moderate (51–70%) or poor (≤ 50%) quality [35]. In cases of disagreement, a consensus was reached in consultation with a third author (JLO).

### Strength of Evidence Synthesis

To determine the strength of evidence of a biomechanical risk factor associated with ACL injuries across various maturity stages in female athletes, the classification system proposed by van Tulder et al. [36] was used:

*Strong evidence* Consistent findings across a minimum of two high-quality studies.

*Moderate evidence* Consistent findings across multiple studies of including at least one high-quality study.

*Limited evidence* One high-quality or multiple moderate-quality or low-quality studies.

*Very limited* One moderate-quality or low-quality study.

*Conflicting evidence* Inconsistent findings across multiple studies.

The findings were rated as consistent and inconsistent when ≥ 75 or < 75% of the studies reported consistent directionality in the findings, respectively [37]. All studies that reported a particular kinematic or kinetic factor were considered in determining the consistency of the findings, irrespective of the study quality. The directionality of the findings was based on the mean values for each biomechanical variable reported across various stages of maturity irrespective of the level of significance (*p* < 0.05).

## Results

### Search Results and Selection

**Fig. 1:**
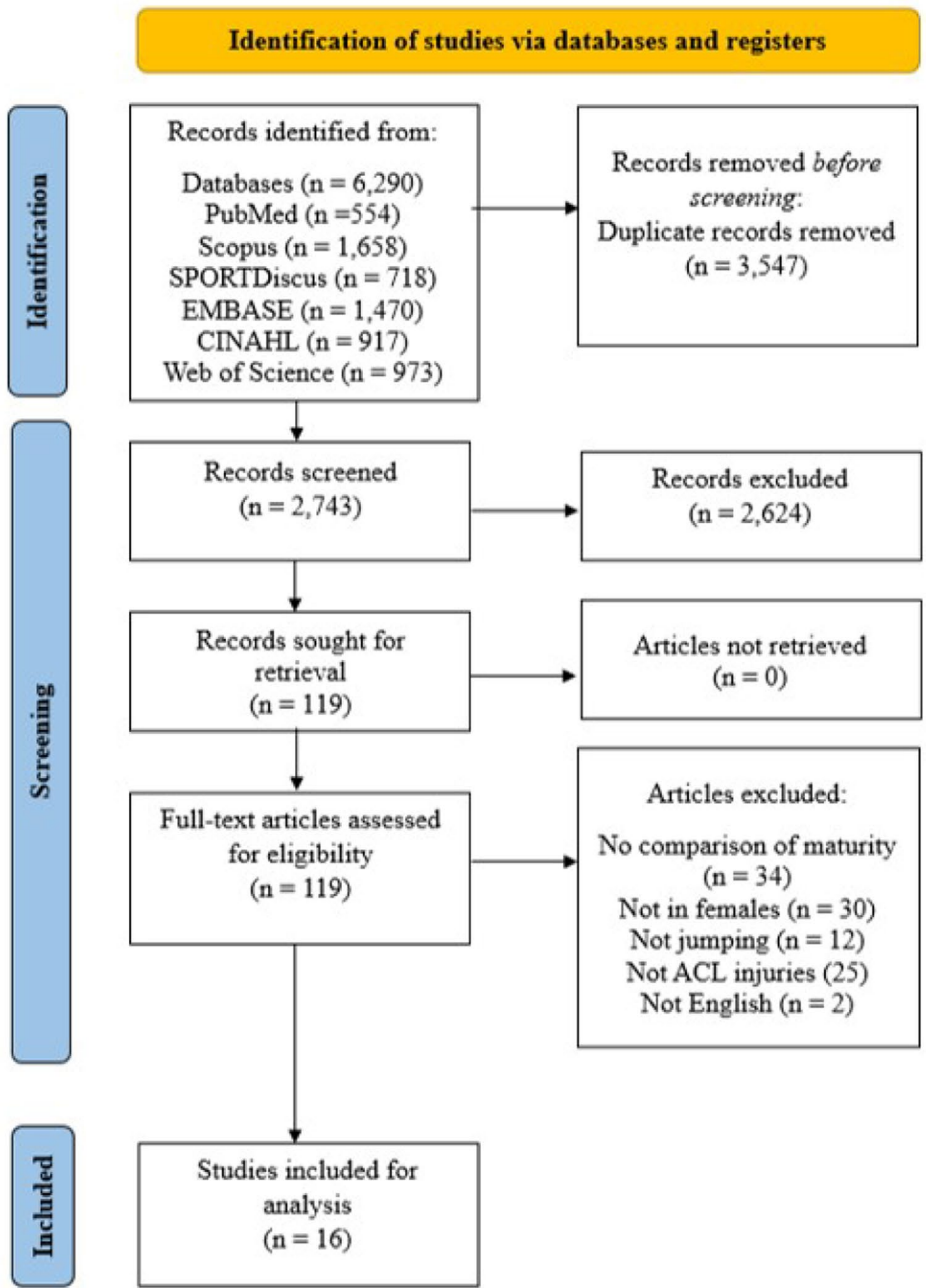
Preferred Reporting Items for Systematic Review and Meta-analyses (PRISMA) flowchart describing the study selection process. *ACL* anterior cruciate ligament

The electronic database and manual search yielded 6290 articles, from which a total of 2743 articles remained after the removal of duplicate studies and 2624 articles were then excluded based on title and abstract screening. Subsequently, a total of 119 full-text articles were screened from which 16 studies [21, 24, 25, 27, 38–49] met full inclusion criteria in our review. The complete search strategy has been detailed in Fig. 1.

### Study Characteristics

In total, 2323 (623 pre-pubertal, 745 pubertal and 955 post-pubertal) female athletes were included in our review. Two studies had classified participants according to their maturity level but did not provide the information regarding the sample size in each maturity group [21, 42], although a combined total of 761 female athletes within the age range of 10–18 years were included in these studies. Based on the data reported from 14 studies, the mean age, stature and mass of the pre-pubertal girls were 10.4 ± 1.2 years, 143.9 ± 7.6 cm and 37.9 ± 5.6 kg; pubertal female girls were 12.5 ± 0.7, 157 ± 4.1 cm and 47.3 ± 4.3 kg; post-pubertal female individuals were 16.7 ± 3 years, 165 ± 2.7 and 59.4 ± 3.4 kg. The maturity level of the athletes was determined using the Pubertal Maturational Observation Scale (PMOS) [38, 39, 43], modified PMOS [24, 25, 27, 40, 42, 45], percentage predicted adult height (PAH) [21, 49], self-assessed Tanner Scale [44], Tanner 5 stages of maturity [46], Tanner grouping [48] and onset of menarche [41]. In two studies that used percentage adult stature for determining maturity status, post-pubertal female individuals were reported to be > 91% of adult stature in one study [21] and > 94% of adult stature in the other study [49]. Across the various scales used in the included studies for estimating the maturity status, the lowest values were used for classifying pre-pubertal girls (i.e. < 85% PAH, Tanner Stage 1, PMOS stage 1), highest for post-pubertal female individuals (i.e. > 95% PAH, Tanner Stages 4–5, PMOS stage 5) and the values in-between for pubertal girls (i.e. 85–95% PAH, Tanner Stages 2–4, PMOS stages 2–4).

The studies included in our review assessed the biomechanical risk factors for ACL injuries during various jump-landing tests. Twelve studies had the participants perform the drop vertical jump [21, 24, 25, 38–43, 46, 47, 49] (DVJ), one study used the SL vertical stop jump test [44], one study used the maximum vertical jump test [48], while two studies had the participants perform the SL drop lateral jump [41, 45] and one study performed the drop jump with a static landing sequence [49]. With respect to the DVJ test, the height from which the participants were asked to jump was predominantly 31 cm [21, 24, 25, 27, 38–40, 42, 43, 49]; however, 30 cm [46] and 36 cm [47] were also used, while the drop height was not reported in one study [41]. Kinematic data were collected using three-dimensional motion capture systems in nine studies [21, 24, 25, 38–40, 44, 48, 49], while two studies used two-dimensional video cameras [41, 46]. Overall, eight studies compared changes in biomechanical variables across all three maturity groups (pre-pubertal, pubertal and post-pubertal) [25, 39, 42, 43, 45–47, 49], four studies compared only pubertal and post-pubertal female individuals [21, 24, 27, 40], and four studies compared pre-pubertal and post-pubertal female athletes [38, 41, 44, 48]. Ten studies were cross-sectional in nature [25, 39, 41, 42, 44–49], four were longitudinal [21, 27, 38, 43] and two had nested cohort [24, 40] study designs. Further details regarding the participant characteristics for all the individual studies have been provided in Table 1.

**Table 1:** Participant characteristics of the included studies

Study	Study design	Sample size	Age (y)	Height (cm)	Weight (kg)	Maturity scale	Stage of maturity	Sporting activity	Sporting level
DiCesare et al. [38]	Prospective/longitudinal	Pre (SS): 79 Pre (MS): 79 Post (SS): 79 Post (MS): 79	Pre- (SS): 12.2 ± 0.8 Pre- (MS): 12.3 ± 0.9 Post (SS): 14.3 ± 1.2 Post (MS): 14.2 ± 1.1	Pre (SS): 155 ± 7.7 Pre (MS): 155 ± 7.5 Post (SS): 160 ± 5.2 Post (MS): 160 ± 5.9	Pre (SS): 47.1 ± 10.1 Pre (MS): 47.6 ± 10.7 Post (SS): 55 ± 7.5 Post (MS): 55.7 ± 8.7	PMOS	Pre: Tanner stage 1 Pub: Tanner stages 2–4 Post: Tanner stage 5	Basketball, soccer and volleyball	SS or MS
Hewett et al. [21]	Longitudinal/cross-sectional	674	10–18	Growing female individuals: 143.6–159.9 Adults: 161.2–165.3	Growing female individuals: 36.3–53.9 Adults: 54.6–61.6	Adult stature	Growing: < 91% stature Adult: > 91% stature	Basketball and soccer	Middle and high school athletes
Quatman et al. [27]	Longitudinal prospective	Pub: 16 Post: 16	Pub: 12.6 ± 1 Post: 13.6 ± 1	Pub: 162 ± 7.9 Post: 165.7 ± 8.4	Pub: 47.5 ± 6 Post: 53.2 ± 6.2	Modified PMOS	Pub: ~ Tanner stages 2 and 3 Post: ~ Tanner stages 4 and 5	Primary: basketball Secondary: track, soccer, softball, baseball, American Football, tennis, swimming and golf	Middle and high-school athletes
Hewett et al. [25]	Cross-sectional	Pre: 14 Pub: 28 Post: 58	Pre: 11.5 ± 0.7 Pub: 12.6 ± 1.1 Post: 15.5 ± 1.5	Pre: 148.7 ± 5.9 Pub: 158.5 ± 6.1 Post: 168.3 ± 6.5	Pre: 38.9 ± 5.9 Pub: 46.8 ± 5.5 Post: 63.4 ± 10.9	Modified PMOS	Pre: Tanner stage 1 Pub: Tanner stages 2 and 3 Post: Tanner stages 4 and 5	Basketball and soccer	Middle and high school athletes
DiStefano et al. [39]	Cross-sectional	Pre: 15 Pub: 12 Post: 27	Pre: 9 ± 1 Pub: 12 ± 3 Post: 16 ± 2	NR	NR	PMOS	Pre: < stage 2 Pub: stages 2–5 Post: > stage 5	NR	NR
Ford et al. [24]	Nested cohort	Pub: 145 Post: 120	Pub: Year 1:12.3 ± 0.8 Year 2: 13.3 ± 0.8 Post: Year 1:14.4 ± 1.4 Year 2: 15.4 ± 1.4	Pub: Year 1:155.9 ± 6.8 Year 2: 160.7 ± 5.9 Post: Year 1:164.4 ± 5.8 Year 2: 165.2 ± 5.8	Pub: Year 1:47.8 ± 10.2 Year 2: 52.7 ± 9.9 Post: Year 1:59 ± 8.5 Year 2: 60.9 ± 8.7	Modified PMOS	Tanner stages 2–5	Basketball and soccer	NR
Ford et al. [40]	Nested cohort	Pub: 182 Post: 133	Pub: Year 1:12.3 ± 0.8 Year 2: 13.3 ± 0.8 Post: Year 1:14.4 ± 1.4 Year 2: 15.4 ± 1.4	Pub: Year 1: 155.9 ± 6.8 Year 2: 160.7 ± 5.9 Post: Year 1: 164.4 ± 5.8 Year 2: 165.2 ± 5.8	Pub: Year 1: 47.8 ± 10.2 Year 2: 52.7 ± 9.9 Post: Year 1: 59 ± 8.5 Year 2: 60.9 ± 8.7	Modified PMOS	Tanner stages 2–5	Basketball and, soccer	NR
Hass et al. [41]	Cross-sectional	Pre: 16 Post: 16	Pre: 9 ± 1 Post: 20.2 ± 1.2	Pre: 134.5 ± 9.1 Post: 162.6 ± 6.1	Pre: 33.1 ± 9.2 Post: 58.5 ± 7.2	Based on menarche	Pre: pre-onset of menarche Post: at least 6 y past the onset of menarche with a normal menstrual cycle	Basketball, soccer, volleyball, gymnastics and dance	Recreational
Hewett et al. [42]	Cross-sectional	87	NR	NR	NR	Modified PMOS	Tanner stages 2–5	NR	Middle and high school athletes
Pedley et al. [43]	Prospective/longitudinal	Pre: 279 Pub: 401 Post: 333	Pre: 11.9 ± 0.6 Pub: 12.5 ± 1.1 Post: 14.8 ± 1.6	Pre: 149.1 ± 6.4 Pub: 158.2 ± 7.1 Post: 164.4 ± 7.5	Pre: 40.1 ± 8.1 Pub: 51.2 ± 10.4 Post: 60.6 ± 10.4	PMOS	Pre: Tanner stage 1 Pub: Tanner stages 2–4 Post: > Tanner stage 5	Basketball, soccer and volleyball	Middle and high-school athletes
Pletcher et al. [44]	Cross-sectional	Pre: 38 Post: 40	Pre: 10.5 ± 0.6 Post: 16.6 ± 0.67	Pre: 148 ± 8 Post: 167 ± 8	Pre: 40.2 ± 10.2 Post: 61.57 ± 10.33	Self-assessed Tanner Scale stage	Pre- (stage 1): school-aged athletes Post- (stage 4): high-school aged athletes	Sports requiring jumping, cutting and landing	School-aged and high-school athletes
Sayer et al. [45]	Cross-sectional	Pre: 31 Pub: 31 Post: 31	Pre: 9.4 ± 1.2 Pub: 11.1 ± 1.4 Post: 19.8 ± 4	Pre: 140 ± 10 Pub: 150 ± 10 Post: 170 ± 10	Pre: 30 ± 5.7 Pub: 38.4 ± 7.4 Post: 60.5 ± 8.5	Modified PMOS	Pre: Tanner stage 1 Pub: Tanner stages 2 and 3 Post: Tanner stages 4 and 5	> 30 min of moderate and/or vigorous activities daily	Recreationally active
Schmitz et al. [46]	Cross-sectional	Pre: 25 Pub: 28 Post: 25	Pre: 10.9 ± 1.4 Pub: 13.5 ± 1.6 Post: 17.6 ± 1	Pre: 154 ± 31.5 Pub: 159.8 ± 8.9 Post: 167.3 ± 6.8	Pre: 40.4 ± 10.2 Pub: 50.2 ± 8.7 Post: 64.9 ± 8.2	Tanner 5 stages of maturity	Pre: Tanner stages 1 and 2 Pub: Tanner stages 3 and 4 Post: Tanner stage 5	NR	Youth and collegiate sports leagues
Sigward et al. [47]	Cross-sectional	Pre: 15 Pub: 15 Post: 14 Adults: 15	Pre: 10.2 ± 0.8 Pub: 12.5 ± 0.7 Post: 15.7 ± 1.1 Adults: 19.3 ± 1.1	Pre: 144.9 ± 7.2 Pub: 156.6 ± 6.8 Post: 166.3 ± 6.7 Adults: 166.1 ± 5.7	Pre: 37.3 ± 6.4 Pub: 47.8 ± 8.9 Post: 59.7 ± 6.8 Adults: 64.9 ± 6.9	Modified PMOS	Pre: Tanner stage 1 Pub: Tanner stages 2–4 Post: Tanner stage 5 Young adult: age > 18 y	Soccer	Club or collegiate level
Swartz et al. [48]	Cross-sectional	Pre: 15 Post: 14	Pre: 9.2 ± 1 Post: 24.2 ± 2.3	Pre: 136.67 ± 6.15 Post: 163.54 ± 6.22	Pre: 32.91 ± 8.1 Post: 62.37 ± 9.11	Tanner grouping	Pre: 7–10 y Post: 19–29 y	Basketball, volleyball, gymnastics	Recreationally active
Westbrook et al. [49]	Cross-sectional	Pre: 17 Pub: 32 Post: 90	Pre: 10.3 ± 0.6 Pub: 11.9 ± 0.8 Post: 14.6 ± 1.6	Pre: 137.8 ± 6.8 Pub: 151.1 ± 5.7 Post: 162.6 ± 5.6	Pre: 34.2 ± 4.5 Pub: 43.3 ± 6 Post: 56.2 ± 8.8	Percent adult stature	Pre: < 87% adult stature Pub: 87–94% adult stature Post: > 94% adult stature	Soccer	NR

*min* minutes, MS multi-sport, *NR* not reported, *PMOS* pubertal maturational observational scale, *Post* post-pubertal, *Pre* pre-pubertal, *Pub* pubertal, *SS* sports specialised, *y* years

### Methodological Quality

The overall methodological quality (mean ± standard deviation) for the included studies was 70 ± 8%, with a range from 53 to 89%. Nine studies [21, 27, 38, 42, 43, 45–48] were classified to be of high quality and seven studies [24, 25, 39–41, 44, 49] were of moderate quality. The detailed scoring for each study can be found in Table 2. Additionally, the criteria used for scoring each question of the Downs and Black checklist can be found in Table S1 of the ESM.

**Table 2:** Quality rating of the included studies as per the Downs and Black checklist

Study	Reporting							External validity		Internal validity				Internal validity-confounding (selection bias)			Power	Results		
	1	2	3	5	6	7	10	11	12	15	16	18	20	21	22	25	27	Total score	Overall rating (%)	Quality
DiCesare et al. [38]	1	1	1	1	1	1	1	1	1	0	1	1	1	0	0	1	0	13/18	72	High
Hewett et al. [21]	1	1	1	1	1	1	1	1	0	0	1	1	1	1	0	1	0	13/18	72	High
Quatman et al. [27]	1	1	1	1	1	1	1	1	1	0	1	1	2	1	1	1	0	16/18	89	High
Hewett et al. [25]	1	1	1	1	1	1	0	1	0	0	1	1	2	0	0	1	0	12/18	67	Moderate
DiStefano et al. [39]	1	1	0	1	1	1	1	0	0	0	1	1	1	0	0	1	0	10/18	56	Moderate
Ford et al. [24]	1	1	1	1	1	1	1	1	0	0	1	1	1	0	0	1	0	12/18	67	Moderate
Ford et al. [40]	1	1	1	1	1	1	1	1	0	0	1	1	1	0	0	1	0	12/18	67	Moderate
Hass et al. [41]	1	1	0	1	1	1	1	1	0	0	1	1	1	0	0	1	0	11/18	61	Moderate
Hewett et al. [42]	1	1	1	1	1	1	1	1	0	0	1	1	1	1	0	1	0	13/18	72	High
Pedley et al. [43]	1	1	1	1	1	1	1	1	0	0	1	1	0	1	0	1	1	13/18	72	High
Pletcher et al. [44]	1	1	1	1	1	1	1	1	0	0	1	1	1	0	0	1	0	12/18	67	Moderate
Sayer et al. [45]	1	1	1	1	1	1	0	1	0	0	1	1	1	1	0	1	1	13/18	72	High
Schmitz et al. [46]	1	1	0	1	1	1	1	1	0	0	1	1	1	1	1	1	0	13/18	72	High
Sigward et al. [47]	1	1	1	1	1	1	1	0	0	0	1	1	1	1	1	1	0	13/18	72	High
Swartz et al. [48]	1	1	1	1	1	1	1	1	0	0	1	1	1	1	0	1	1	14/18	78	High
Westbrook et al. [49]	1	1	1	1	1	1	1	0	0	0	1	1	1	0	0	1	0	11/18	61	Moderate

### Strength of Evidence Synthesis

**Fig. 2:**
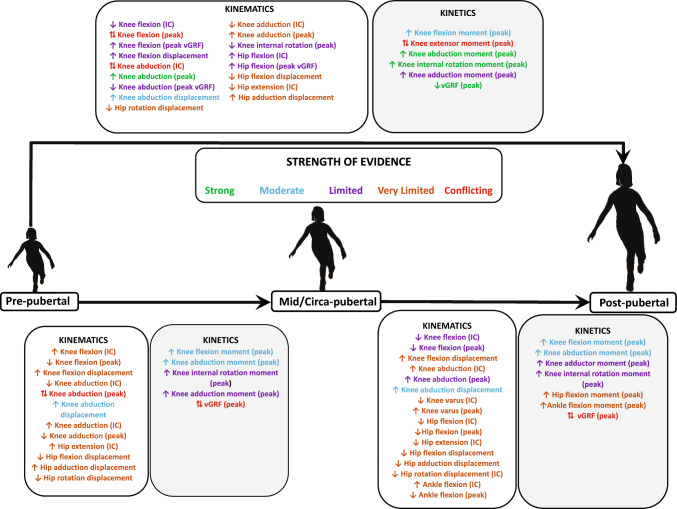
Summary of the strength of evidence for the kinematic and kinetic variables across various stages of maturity. *IC* initial contact, *vGRF* ground reaction force, ↑ indicates higher kinematic or kinetic values as female athletes progress to the next stage of maturity, ↓ indicates lower kinematic or kinetic values as female athletes progress to the next stage of maturity, ↔ indicates similar kinematic and kinetic values as female athletes progress to the next stage of maturity, ↑↓ indicates conflicting evidence

For the kinematic variables, we found strong evidence for post-pubertal female individuals having a higher peak knee abduction angle compared with pre-pubertal and pubertal girls. With regard to the kinetic variables, we found strong evidence for post-pubertal female individuals having higher knee abduction and internal rotation moments compared with pre-pubertal girls. There was strong evidence for pre-pubertal girls having higher peak relative vGRF (normalised to BW) compared with post-pubertal female individuals. The strength of evidence for all the other kinematic and kinetic variables ranged from moderate to conflicting and could not be determined in some instances. Table 3 and Fig. 2 provide the detailed results of the findings.

**Table 3:**
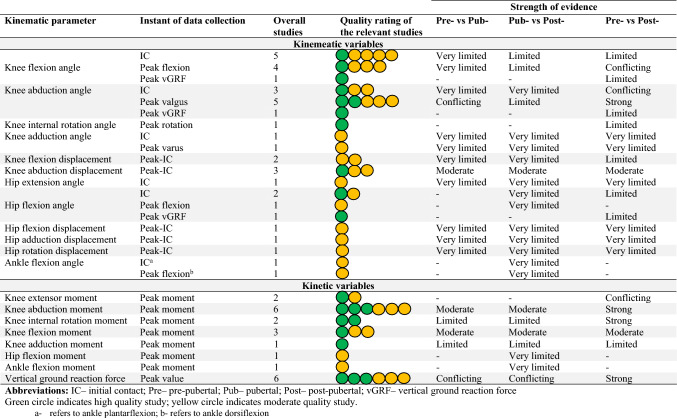
Strength of evidence for the kinematic and kinetic variables reported in the included studies

Green circle indicates a high-quality study; yellow circle indicates a moderate-quality study

*IC* initial contact, post- post-pubertal, *Pre-* pre-pubertal, *Pub*- pubertal, *vGRF* vertical ground reaction force

^a^Ankle plantarflexion

^b^Ankle dorsiflexion

### Kinematic Risk Factors

A total of 12 kinematic variables were identified across the included studies. The findings of the kinematic variables from the individual studies are summarised in Table 4.

**Table 4:** Kinematic variables at different instances of foot contact during jump landing tasks reported in the included studies

Study	Jumping test performed	Instant of kinematic data	Temporal phase of the jump analysed	Unit of measure	Pre- athletes	Pub athletes	Post- athletes	Maturational relationship	Study findings
**Knee flexion angle**									
DiCesare et al. [38]	DVJ (31-cm box)	Peak	**Stance phase:** IC (vGRF > 10 N) to toe-off (vGRF < 10 N)	Degree	Dominant (SS): 83.2 ± 8.2 Non-dominant (SS): 84.2 ± 8.5 Dominant (MS): 82.9 ± 9.2 Non-dominant (MS): 84.4 ± 9.6	–	Dominant (SS): 82.1 ± 9 Non-dominant (SS): 83.3 ± 9 Dominant (MS): 82.7 ± 9.6 Non-dominant (MS): 84.2 ± 9.7	Pre- > Post-	MS: NS: *p* > 0.05 SS: NS: *p* > 0.05
DiStefano et al. [39]	DVJ (31-cm box)	IC	Stance phase: IC (vGRF > 10 N) to toe-off (vGRF < 10 N)	Degree	32.78 ± 6.3	33.82 ± 11.38	24.76 ± 5.75	Pub > Pre- > Post-	S: p < 0.05
Ford et al. [24]	DVJ (31-cm box)	IC, peak	**Stance phase:** IC to lowest vertical position of COM	Degree	–	IC: Year 1: 22.9 ± 7.4 Year 2: 22.6 ± 7.2 Peak: Year 1: 83.1 ± 8.9 Year 2: 83.9 ± 9.1	IC: Year 1: 22.0 ± 6.8 Year 2: 21.8 ± 5.9 Peak: Year 1: 81.2 ± 8.1 Year 2: 81.9 ± 7.7	IC: Pub > Post- Peak flexion: Pub > Post-	IC: NS: *p* > 0.05 Peak flexion: NS: *p* > 0.05
Hass et al. [41]	DVJ with static, vertical and lateral landing	Average at IC	**Stance phase:** 100 ms before IC to 500 ms after contact	Degree	20 ± 1	–	15 ± 1	Pre- > Post-	S: *p* < 0.005
Pletcher et al. [44]	SL vertical stop jump	IC, peak	Stance phase	Degree	IC: 13.89 ± 7.09 Peak flexion: 37.39 ± 10.68	–	IC: 12.54 ± 6.62 Peak flexion: 41.41 ± 9.27	IC: Pre- > Post- Peak flexion: Post- > Pre-	IC: NS: *p* = 0.559 Peak flexion: NS: *p* = 0.619
Swartz et al. [48]	Maximum vertical jump	IC, peak vGRF	Stance phase	Degree	IC: 10.7 ± 7.18 Peak vGRF: 31.5 ± 6.17	–	IC: 11.56 ± 6.24 Peak vGRF: 38 ± 9.52	IC: Post- > Pre- Peak vGRF: Post- > Pre-	IC: NR Peak vGRF: NR
Westbrook et al. [49]	DVJ (31-cm box)	Peak	**Stance phase:** IC (vGRF > 10 N) to toe-off (vGRF < 10 N)	Degree	85.7 ± 8.7	82.7 ± 11.1	82.2 ± 9.7	Pre- > Pub > Post-	S: *p* < 0.001
**Knee abduction angle**									
DiCesare et al. [38]	DVJ (31-cm box)	Peak	**Stance phase:** IC (vGRF > 10 N) to toe-off (vGRF < 10 N)	Degree	Dominant (SS): 8.9 ± 6.6 Non-dominant (SS): 10.1 ± 6.2 Dominant (MS): 7.6 ± 4.8 Non-dominant (MS): 8.2 ± 5.5	–	Dominant (SS): 11.3 ± 6.7 Non-dominant (SS): 11.6 ± 5.1 Dominant (MS): 8.6 ± 5.3 Non-dominant (MS): 9.5 ± 5.1	Post- > Pre-	SS: *p* = 0.005 MS: *p* > 0.05
Hewett et al. [21]	DVJ (31-cm box)	Peak	**Stance phase:** IC (vGRF > 10 N) to toe-off (vGRF < 10 N)	Degree	7.4–10.5	–	8.9–10.4	Post- > Pre-	S: *p* = 0.001
Hewett et al. [25]	DVJ (31-cm box)	IC, peak	Stance phase	Degree	IC: 1.6 ± 1.5 Peak valgus: 13.9 ± 4.4	IC: 0.8 ± 1.1 Peak valgus: 19.7 ± 3.1	IC: 4.7 ± 0.7 Peak valgus: 29.6 ± 2.7	IC: Post- > Pre- > Pub Peak valgus: Post- > Pub > Pre-	IC: S: *p* < 0.05 Peak valgus: S: *p* < 0.05
Ford et al. [40]	DVJ (31-cm box)	Peak	**Stance phase:** IC to lowest vertical position of COM	Degree	–	Year 1: 7.7 ± 6.1 Year 2: 9.3 ± 6.2	Year 1: 9.3 ± 5.7 Year 2: 9.4 ± 5.6	Post- > Publ	S: *p* < 0.05
Pletcher et al. [44]	SL vertical stop jump	IC	Stance phase	Degree	2.48 ± 7.78	–	2.95 ± 4.41	Post- > Pre-	NS: *p* = 0.369
Swartz et al. [48]	Maximum vertical jump	IC, peak vGRF	Stance phase	Degree	IC: 11.67 ± 4.38 Peak vGRF: 9.63 ± 4.73	–	IC: 9.75 ± 2.7 Peak vGRF: 7.68 ± 4	IC: Pre- > Post- Peak vGRF: Pre- > Post-	IC: NR Peak vGRF: NR
Westbrook et al. [49]	DVJ (31-cm box)	Peak	**Stance phase:** IC (vGRF > 10 N) to toe-off (vGRF < 10 N)	Degree	8.4 ± 6.6	6.4 ± 5.8	10.5 ± 6.3	Post- > Pre- > Pub	S: *p* < 0.001
**Knee internal rotation angle**									
DiCesare et al. [38]	DVJ (31-cm box)	Peak	**Stance phase:** IC (vGRF > 10 N) to toe-off (vGRF < 10 N)	Degree	Dominant (SS): 6.8 ± 5.7 Non-dominant (SS): 7.6 ± 5.1 Dominant (MS): 7.0 ± 5.8 Non-dominant (MS): 8.4 ± 5.7	–	Dominant (SS): 5.6 ± 4.4 Non-dominant (SS): 6.8 ± 5.7 Dominant (MS): 6.5 ± 6.1 Non-dominant (MS): 7.9 ± 5.3	Pre- > Post-	MS: NS: *p* > 0.05 SS: NS: *p* > 0.05
**Knee adduction angle**									
DiStefano et al. [39]	DVJ (31-cm box)	IC, peak	**Stance phase:** IC (vGRF > 10 N) to toe-off (vGRF < 10 N)	Degree	IC: 2.50 ± 4.70 Peak varus: 10.70 ± 6.06	IC: 3.81 ± 10.93 Peak varus: 10.45 ± 12.10	IC: 1.92 ± 5.10 Peak varus: 12.32 ± 7.76	IC: Pub > Pre- > Post- Peak varus: Post- > Pre- > Pub	IC: NS: *p* > 0.05 Peak varus: NS: *p* > 0.05
**Knee flexion displacement**									
DiStefano et al. [39]	DVJ (31-cm box)	Peak, IC	**Stance phase:** IC (vGRF > 10 N) to toe-off (vGRF < 10 N)	Degree	58.95 ± 11.74	61.20 ± 12.94	62.59 ± 10.92	Post- > Pub > Pre-	NS: *p* > 0.05
Hass et al. [41]	DVJ with static, vertical and lateral landing	Change in angle during landing phase	**Stance phase:** 100 ms before IC to 500 ms after contact	Degree	43 ± 2	–	46 ± 1	Post- > Pre-	NS: *p* > 0.05
**Knee abduction displacement**									
Hewett et al. [25]	DVJ (31-cm box)	Unitless	Stance phase	cm	0.029 ± 0.005	0.034 ± 0.003	0.042 ± 0.003	Post- > Pub > Pre-	S: *p* < 0.05
Hass et al. [41]	DVJ with static, vertical and lateral landing	Change in angle during landing phase	**Stance phase:** 100 ms before IC to 500 ms after contact	Degree	2 ± 1	–	4 ± 1	Post- > Pre-	NS: *p* > 0.05
Schmitz et al. [46]	DVJ (30-cm box)	IC, take-off	IC to take-off (visual inspection)	Degree	LS: 10 ± 6.4 RS: 12.9 ± 7.4	LS: 12.2 ± 8.1 RS: 13.5 ± 9.6	LS: 15.9 ± 9.2 RS: 15.1 ± 8.4	Post- > Pub > Pre-	S: *p* < 0.05
**Hip extension angle**									
DiStefano et al. [39]	DVJ (31-cm box)	IC	**Stance phase:** IC (vGRF > 10 N) to toe-off (vGRF < 10 N)	Degree	32.64 ± 9.03	33.38 ± 10.35	27.37 ± 9.24	Pub > Pre- > Post-	NS: *p* > 0.05
**Hip flexion angle**									
Ford et al. [24]	DVJ (31-cm box)	IC, peak	**Stance phase:** IC to lowest vertical position of COM	Degree	–	IC: Year 1: 26.9 ± 8.1 Year 2: 27.4 ± 7.1 Peak: Year 1: 56.6 ± 9.2 Year 2: 57.7 ± 8.3	IC: Year 1: 26.9 ± 8.1 Year 2: 26.8 ± 6.8 Peak: Year 1: 56.1 ± 8.9 Year 2: 57.3 ± 8.9	IC: Pub > Post- Peak flexion: Pub > Post-	IC: NS: *p* > 0.05 Peak flexion: NS: *p* > 0.05
Swartz et al. [48]	Maximum vertical jump	IC, peak vGRF	Stance phase	Degree	IC: 7.12 ± 5.22 Peak vGRF: 12.4 ± 5.18	–	IC: 14.09 ± 5.12 Peak vGRF: 21.49 ± 7.01	IC: Post- > Pre- Peak vGRF: Post- > Pre-	IC: NR Peak vGRF: NR
**Hip flexion displacement**									
DiStefano et al. [39]	DVJ (31-cm box)	Peak, IC	**Stance phase:** IC (vGRF > 10 N) to toe-off (vGRF < 10 N)	Degree	39.52 ± 7.23	33.03 ± 12.85	31.98 ± 14.77	Pre- > Pub > Post-	NS: *p* > 0.05
**Hip adduction displacement**									
DiStefano et al. [39]	DVJ (31-cm box)	Peak, IC	**Stance phase:** IC (vGRF > 10 N) to Toe-off (vGRF < 10 Nt	Degree	14.84 ± 3.85	16.44 ± 4.69	15.60 ± 6.01	Pub > Post- > Pre-	NS: *p* > 0.05
**Hip rotation displacement**									
DiStefano et al. [39]	DVJ (31-cm box)	Peak, IC	**Stance phase:** IC (vGRF > 10 N) to toe-off (vGRF < 10 N)	Degree	13.41 ± 6.65	11.66 ± 6.40	10.98 ± 6.29	Pre- > Pub > Post-	NS: *p* > 0.05
**Ankle flexion angle**									
Ford et al. [24]	DVJ (31-cm box)	IC, peak	**Stance phase:** IC to lowest vertical position of COM	Degree	–	IC^a^: Year 1: 24.0 ± 6.8 Year 2: 24.6 ± 6.8 Peak^b^: Year 1: 30.3 ± 4.9 Year 2: 30.1 ± 5.1	IC^a^: Year 1: 24.9 ± 6.1 Year 2: 24.9 ± 5.7 Peak^b^: Year 1: 29.1 ± 4.8 Year 2: 29.3 ± 4.6	IC^a^: Post- > Pub Peak flexion^b^: Pub > Post-	NS: *p* > 0.05

COM centre of mass, *DVJ* drop vertical jump, *IC* initial contact, *LS* left side, *MS* multi-sport, *NR* not reported, *NS* non-significant, *Post-* post-pubertal, *Pre-* pre-pubertal, *Pub* pubertal, *RS* right side, *S* significant, *SL* single leg, *SS* sports-specialised, *vGRF* vertical ground reaction force

^a^Ankle plantarflexion

^b^Ankle dorsiflexion

#### Knee Kinematics

Seven studies reported variables associated with the knee flexion angle during various jump-landing tests, including the knee angle at IC, peak knee flexion and knee flexion at peak vGRF. Post-pubertal female individuals had the lowest knee flexion at IC in three studies [24, 39, 41, 44] of moderate quality and the highest knee flexion at IC in one high-quality study [48] when compared with pre-pubertal girls. Two moderate-quality studies [24, 39] reported post-pubertal female individuals to have the lower knee flexion angle at IC compared with pubertal girls. One moderate-quality study [39] reported pre-pubertal girls to have lower knee flexion at IC compared with pubertal girls.

Knee flexion angle at peak knee flexion was reported in four studies, with variable findings [24, 38, 44, 49]. The high-quality study by DiCesare et al. [38] reported post-pubertal female individuals to have a lower peak knee flexion angle compared with pre-pubertal girls. Other studies of moderate quality showed equivocal findings, with higher peak knee flexion in pre-pubertal [49] or post-pubertal [44] groups. Two studies of moderate quality found post-pubertal female individuals to have lower peak knee flexion compared with pubertal girls [24, 49]. For knee flexion angle at peak vGRF, pre-pubertal girls were found to have lower values than post-pubertal female individuals [48].

Knee abduction angle during jump-landing tasks was reported in seven studies. A high-quality study by Swartz et al. [48] reported pre-pubertal girls to have higher knee abduction compared with post-pubertal peers. However, contradictory findings were reported in two studies of moderate quality [25, 44], in which pubertal girls were found to have the highest knee abduction angle at IC compared with pre-pubertal and post-pubertal girls in one moderate-quality study.

Five studies reported data for the peak knee abduction angle [21, 25, 38, 40, 49], with varying quality of evidence showing post-pubertal female individuals had higher peak knee abduction compared with pre-pubertal and pubertal girls. A high-quality study by Swartz et al. [48] reported pre-pubertal girls to have a higher peak knee abduction angle compared with post-pubertal peers at the instant of peak vGRF. Knee abduction displacement was reported in two studies of high [46] and moderate quality [25], with post-pubertal athletes displaying consistently higher values compared with pubertal and pre-pubertal girls.

Several other knee-related kinematic variables were reported by two studies of moderate [39] and poor [41] quality. Post-pubertal female individuals had higher knee flexion [39, 52] and knee abduction displacement [41] compared with pubertal and pre-pubertal girls, with the exception of the knee adduction angle at IC, which was higher in pubertal girls [39].

#### Hip Kinematics

Hip flexion angle at IC and peak vGRF [24, 48], hip extension angle at IC [39], and hip flexion, adduction and rotation displacement [39] were reported in several individual studies. These studies were of high [48] and moderate [24] quality. Pre-pubertal girls had lower hip flexion at IC and peak vGRF compared with pubertal and post-pubertal female individuals [47, 56], with the highest values reported in pubertal female individuals [47]. Pubertal and post-pubertal female individuals had lower hip flexion and angular displacement than pre-pubertal girls [39]. Pubertal girls had higher hip adduction displacement and extension at IC than pre-pubertal and post-pubertal girls [39].

#### Ankle Kinematics

One study reported the ankle plantarflexion and dorsiflexion angle, which showed that post-pubertal female individuals had a higher ankle plantarflexion at IC, but a lower peak ankle dorsiflexion, compared with pubertal girls [24].

### Kinetic Risk Factors

A total of eight kinetic variables were identified across the included studies. The moments reported for the hip, knee and ankle joints were external moments. The findings of the kinetic variables from the individual studies are summarised in Table 5.

**Table 5:** Kinetic variables at different instances of foot contact during jump landing tasks reported in the included studies

Study	Jumping test performed	Instant of kinetic data	Temporal phase of the jump analysed	Unit of measure	Pre- athletes	Pub athletes	Post- athletes	Direction of the study results	Study findings
**Knee extensor moment**									
DiCesare et al. [38]	DVJ (31-cm box)	Peak moment	**Stance phase:** IC (vGRF > 10 N) to toe-off (vGRF < 10 N)	Nm	Dominant (SS): 89.3 ± 23.6 Non-dominant (SS): 83.8 ± 22.5 Dominant (MS): 87.6 ± 27.1 Non-dominant (MS): 83.4 ± 25	–	Dominant (SS): 107.3 ± 23.4 Non-dominant (SS): 103.1 ± 12.8 Dominant (MS): 112.3 ± 28 Non-dominant (MS): 108.7 ± 24.5	Post > Pre	SS: *p* = 0.032 MS: *p* > 0.05
Hass et al. [41]	DVJ with static, vertical and lateral landing	Peak moment	**Stance phase:** 100 ms before IC to 500 ms after contact	Unitless	0.0124 ± 0.001	–	0.0079 ± 0.001	Pre > Post	S: *p* < 0.05
**Knee abduction moment**									
DiCesare et al. [38]	DVJ (31-cm box)	Peak moment	**Stance phase:** IC (vGRF > 10 N) to toe-off (vGRF < 10 N)	Nm	Dominant (SS): 15.2 ± 11.6 Non-dominant (SS): 19.9 ± 11.1 Dominant (MS): 13.9 ± 9.1 Non-dominant (MS): 18.8 ± 12.8	–	Dominant (SS): 23.8 ± 14.7 Non-dominant (SS): 26.9 ± 12.6 Dominant (MS): 19.6 ± 11.4 Non-dominant (MS): 23 ± 14.2	Post > Pre	SS: *p* = 0.006 MS: *p* > 0.05
Hewett et al. [21]	DVJ (31-cm box)	Peak moment	**Stance phase:** IC (vGRF > 10 N) to Toe-off (vGRF < 10 N)	Nm	12.4–22.6	–	23.6–25.8	Post > Pre	S: *p* < 0.05
Ford et al. [40]	DVJ (31-cm box)	Peak moment	**Stance phase:** IC to lowest vertical position of COM	Nm	–	Year 1: 14.4 ± 10.9 Year 2: 19.2 ± 12.1	Year 1: 20.6 ± 13.7 Year 2: 23.2 ± 13.4	Post > Pub	S: *p* < 0.05
Hass et al. [41]	DVJ with static, vertical and lateral landing	Peak moment	**Stance phase:** 100 ms before IC to 500 ms after contact	Unitless	0.0016 ± 0.002	–	0.0012 ± 0.002	Pre > Post	NS: *p* > 0.05
Sayer et al. [45]	SL drop lateral jump	Peak moment	First 25% of stance phase	Nm/kg	0.34 ± 0.11	0.37 ± 0.13	0.43 ± 0.18*	Post > Pub > Pre	S: *p* < 0.05
Westbrook et al. [49]	DVJ (31-cm box)	Peak moment	**Stance phase:** IC (vGRF > 10 N) to toe-off (vGRF < 10 N)	Nm	13.1 ± 8.6	14.6 ± 9.7	27.3 ± 15	Post > Pub > Pre	S: *p* < 0.001
**Knee internal rotation moment**									
DiCesare et al. [38]	DVJ (31-cm box)	Peak moment	**Stance phase:** IC (vGRF > 10 N) to Toe-off (vGRF < 10 N)	Nm	Dominant (SS): 3 ± 2.8 Non-dominant (SS): 6.4 ± 4.3 Dominant (MS): 3.6 ± 3.5 Non-dominant (MS): 7.2 ± 4.3	–	Dominant (SS): 3.7 ± 4.5 Non-dominant (SS): 6.4 ± 4.8 Dominant (MS): 4.4 ± 4.1 Non-dominant (MS): 7.6 ± 5.1	Post > Pre	MS: NS: *p* > 0.05 SS: NS: *p* > 0.05
Sayer et al. [45]	SL drop lateral jump	Peak moment	First 25% of stance phase	Nm/kg	0.16 ± 0.07	0.18 ± 0.06	0.23 ± 0.10	Post > Pub > Pre	S: *p* < 0.05
**Knee flexion moment**									
Ford et al. [24]	DVJ (31-cm box)	Peak moment	**Stance phase:** IC to lowest vertical position of COM	Nm	–	Year 1: 91.1 ± 24.5 Year 2: 100.4 ± 25.5	Year 1: 113.8 ± 29.2 Year 2: 115.5 ± 28.1	Post > Pub	S: *p* < 0.05
Westbrook et al. [49]	DVJ (31-cm box)	Peak moment	**Stance phase:** IC (vGRF > 10 N) to toe-off (vGRF < 10 N)	Nm	62.6 ± 10.8	98.9 ± 30.6	− 116.8 ± 38.7	Post > Pub > Pre	S: *p* < 0.001
Sayer et al. [45]	SL drop lateral jump	Peak moment	First 25% of stance phase	Nm/kg	2.41 ± 0.41	2.69 ± 0.54	2.86 ± 0.41*	Post > Pub > Pre	S: *p* < 0.05
**Knee adduction moment**									
Sigward et al. [47]	DVJ (36-cm box)	Average moment	**Stance phase:** IC to peak knee flexion	Nm/kg⋅m	0.03 ± 0.024	0.08 ± 0.04	0.097 ± 0.027	Post > Pub > Pre	NS: *p* > 0.05
**Hip flexion moment**									
Ford et al. [24]	DVJ (31-cm box)	Peak moment	**Stance phase:** IC to lowest vertical position of COM	Nm	–	Year 1: 71.5 ± 18.8 Year 2: 80.9 ± 21.0	Year 1: 91.2 ± 22.3 Year 2: 94.1 ± 23.9	Post > Pub	NS: *p* > 0.05
**Ankle dorsiflexion moment**									
Ford et al. [24]	DVJ (31-cm box)	Peak moment	**Stance phase:** IC to lowest vertical position of COM	Nm	–	Year 1: 73.1 ± 20.8 Year 2: 81.6 ± 20.9	Year 1: 91.1 ± 20.7 Year 2: 95.0 ± 22.2	Post > Pub	NS: *p* > 0.05
**vGRF**									
Quatman et al. [27]	DVJ (31-cm box)	Peak vGRF	Stance phase	BW	–	D: 2.2 ± 0.6 ND: 1.9 ± 0.4	D: 2.2 ± 0.4 ND: 1.9 ± 0.5	Pub = Post	NS: *p* = 0.63–0.75
DiStefano et al. [39]	DVJ (31-cm box)	Peak vGRF	**Stance phase:** IC (vGRF > 10 N) to toe-off (vGRF < 10 N)	% BW	1.75 ± 0.33	1.75 ± 0.33	1.67 ± 0.38	Pre = Pub > Post	NS: *p* > 0.05
Hass et al. [41]	DVJ with static, vertical and lateral landing	Peak vGRF	**Stance phase:** 100 ms before IC to 500 ms after contact	Unitless	Toe contact: 3.46 ± 0.23 Heel contact: 7.18 ± 0.40	–	Toe contact: 2.23 ± 0.23 Heel contact: 6.4 ± 0.4	Pre > Post	Toe contact: S: *p* < 0.05 Heel contact: NS: *p* > 0.05
Hewett et al. [42]	DVJ (31-cm box)	Landing phase	Landing to take-off phase	% change	–	5.3–8.4% increase	16.8% decrease	CND	NS: *p* > 0.05
Pedley et al. [43]	DVJ (31-cm box)	Peak vGRF	**Stance phase:** IC (vGRF > 10 N) to toe-off (vGRF < 10 N)	BW	8.71 ± 1.54	8.49 ± 1.64	8.48 ± 1.66	Pre > Pub > Post	NS: *p* > 0.05
Pletcher et al. [44]	SL vertical stop jump	Peak vGRF	Stance phase	% BW	3.63 ± 0.76	–	3.39 ± 0.54	Pre > Post	S: *p* = 0.015
Swartz et al. [48]	Maximum vertical jump	Peak vGRF	Stance phase	N/J	IC: 8.21 ± 2.3	–	IC: 5.28 ± 1.29	Pre > Post	NR

*BW* bodyweight, *CND* could not be determined, *COM* centre of mass, *DVJ* drop vertical jump, *IC* initial contact, *LH* landing height, *MS* multi-sport, *NS* non-significant, *Post-* post-pubertal, *Pre-* pre-pubertal, *Pub* pubertal, *ROM* range of motion, *S* significant, *SL* single leg, *SS* sports-specialised, *vGRF* vertical ground reaction force

*Significant when pre- and post- female individuals were compared

#### Knee Kinetics

Sagittal plane external knee extensor moment was reported in both a high-quality study [38] and a moderate-quality [41] study. While DiCesare et al. [38] reported post-pubertal female individuals had a higher peak knee extensor moment compared with pre-pubertal girls, contradictory findings were reported in the other study [41]. Post-pubertal female individuals had the highest peak external knee flexion moment (i.e. more quadriceps dominant) values followed by pubertal and then pre-pubertal girls [45, 49].

Peak external knee abduction moment during jump-landing tasks was reported in three high-quality studies [21, 38, 45] and three moderate-quality studies [40, 41, 49]. Post-pubertal female individuals had the highest values followed by pubertal and pre-pubertal girls in five studies [21, 24, 38, 45, 49], while pre-pubertal girls had the highest value in one study [41].

Post-pubertal female individuals had the highest peak internal rotation moment values followed by pubertal and pre-pubertal girls. Post-pubertal female individuals also had the highest average knee adductor moment followed by pubertal and pre-pubertal girls.

#### Hip and Ankle Kinetics

Peak external hip flexion and ankle dorsiflexion moments were reported in one moderate-quality study [24], which identified that post-pubertal girls had higher values compared with pubertal girls.

#### Vertical Ground Reaction Force

Vertical ground reaction force normalised to BW during various jump-landing tasks were reported in four high-quality studies [27, 42, 43, 48] and three moderate-quality studies [39, 41, 44]. In addition, a moderate-quality study [42] reported the percentatage change in landing vGRF across various stages of maturity. Overall, pre-pubertal girls were found to have consistently higher values in vGRF compared to post-pubertal female individuals in five studies [39, 41, 43, 44, 48], The findings were equivocal when pre-pubertal girls were compared with pubertal girls. While one high-quality study [43] reported pre-pubertal girls to have marginally higher values, the other study [39] reported the values to be the same in both groups. Similarly, the findings were contradictory when pubertal girls were compared with post-pubertal female individuals. While a high-quality study [43] and a moderate-quality study [39] reported pubertal girls to have higher values, another high-quality study [27] reported the values to be the same in both groups. One study reported changes in vGRF during the landing phase of a jump-landing task [50]. The vGRF in female individuals decreased by 5.3% and then increased by 8.4% from Tanner stages 2 to 3 and then decreased by 16.8% from Tanner stages 3 to 4 and 4 to 5.

## Discussion

**Fig. 3:**
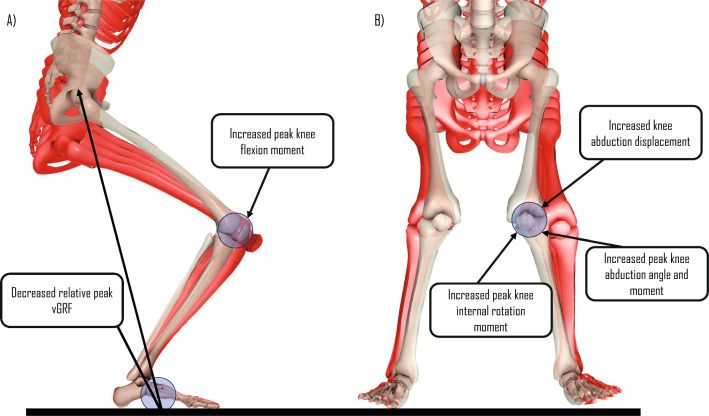
Summary of kinematic and kinetic variables in the **A** sagittal and **B** frontal plane for meaningful changes from pre-pubertal (*red skeletons*) to post-pubertal (*white skeleton*) female individuals. This figure has been adapted from the study published by Galloway et al. [82] with permission from the publisher (License Number: 5746521199753). *vGRF* vertical ground reaction force

Our review aimed to synthesise the changes in potential biomechanical risk factors associated with ACL injuries across various stages of maturity in female athletes. Common scales used for determining the maturity level of female athletes included PMOS, modified PMOS, % predicted adult height, self-assessed Tanner Scale, Tanner 5 stages of maturity, Tanner grouping and onset of menarche. The jump-landing tasks used for reporting the variation in biomechanics across different stages of maturity were DVJ, SL vertical stop jump test, maximum vertical jump, SL drop lateral jump and drop jump with a static landing sequence. Of the 12 kinematic variables reported in our review, we found strong evidence for a higher peak knee abduction angle in post-pubertal female individuals compared with pre-pubertal girls during deceleration tasks. With regard to the eight kinetic variables, we found strong evidence for higher knee abduction and internal rotation moments in post-pubertal female individuals compared with pre-pubertal girls (Fig. 3). There was also strong evidence for higher peak vGRF in pre-pubertal girls compared with post-pubertal female individuals (Fig. 3). The strength of evidence for the remaining kinematic and kinetic variables ranged from conflicting to moderate and, in some instances, could not be determined highlighting the need for further research in this area.

### Knee Abduction

We found strong evidence for a higher peak knee abduction angle in post-pubertal female individuals compared with pre-pubertal athletes. Further, post-pubertal female individuals also had higher knee abduction displacement compared with pre-pubertal athletes. Our findings are similar to previous meta-analytical data in which female individuals were found to have an increased knee abduction angle with increasing maturity [50]. Anterior cruciate ligament load is a result of the combination of higher knee abduction and internal rotation moments [51], and we found strong evidence that these kinetic variables were increased with maturation and peaked in post-pubertal female individuals. It has been speculated that female individuals tend to become more ligament dominant as they mature whereby they employ more limited knee flexion and rely more on frontal place loading to decelerate their centre of mass during maturation [21, 25, 52]. In addition, the rapid increases in lower extremity limb length accompanied by a marked increase in body mass that occur during adolescence, in the absence of sufficient neuromuscular adaptation, are related to decreased dynamic knee stability and increased joint torque loads in female athletes [21]. Interestingly, we found conflicting evidence for the knee abduction angle at IC between pre-pubertal and post-pubertal female individuals and peak knee abduction angle between pre-pubertal and pubertal female individuals. A variety of jump-landing tasks such as the DVJ, SL vertical stop jump and maximum vertical jump tests were used across various studies. Therefore, variation and complexity in the jumping protocol implemented along with differences in sample size (see Table 1), mode of data collection (two dimensional or three dimensional), inadequate training experience and poor technique in performing advanced jumping tests such as SL vertical jumping, and the use of various scales for estimating the maturity status of female athletes (PMOS vs % adult stature) could have led to equivocal findings across the respective studies.

Notably, DiCesare et al. [38] found sport-specialised athletes had a higher knee abduction angle compared with multi-sport athletes; however, higher values were observed in post-pubertal female individuals compared with pre-pubertal girls irrespective of their specialisation status. The literature suggests that diversification of movement should be prioritised during early childhood and adolescence for comprehensive motor and coordination development [53]. Conversely, early sport specialisation may reduce movement variability [54] and promote the development of a narrow range of specialised skills, thereby negatively impacting the development of a child’s motor skills portfolio [55]. Therefore, future research should consider the potential role of sports specialisation on jump-landing biomechanics across various maturity levels and whether it predisposes athletes to a higher risk of ACL injury. Incorporating other training programmes could be beneficial for athletes in order to develop movement competence during various tasks irrespective of their participation in single or multiple sports. For instance, previous research has indicated that neuromuscular training programmes were effective in reducing the risk of ACL injuries in early or mid-teens by 72% (odds ratio: 0.278) [56]. This review included studies that had performed various interventions such as plyometrics, balance training, weight training, and speed and agility training. Another review highlighted that a multi-faceted training intervention with at least three different exercise types and techniques was beneficial in reducing ACL injuries in female athletes under 19 years of age, with plyometric and strength training being the most commonly recommended forms of exercises [57]. In light of these findings, it might be beneficial for female athletes to be exposed to neuromuscular training interventions from a young age (i.e. in the pre-pubertal years) to improve their jump-landing biomechanics and reduce their risk of ACL injury.

### Knee Flexion

We found limited evidence differentiating pre-pubertal and pubertal female athletes for knee flexion angle at IC and conflicting evidence for maturational differences in peak knee flexion during jump-landing tasks. Of the three studies that compared pre-pubertal and post-pubertal female individuals, two studies of high [38] and moderate [49] quality found post-pubertal female individuals had lower peak knee flexion compared with pre-pubertal girls, whereas another study [44] reported the former to have a higher peak knee flexion angle, albeit the findings were non-significant. Interestingly, we also found very limited evidence that pre-pubertal girls had reduced knee flexion range of motion compared with post-pubertal female individuals during SL static, vertical and lateral drop jump-landing tasks [41]. These differences could primarily be attributed to the differences in jump-landing protocols incorporated in the included studies. While DiCesare et al. [38] and Westbrook et al. [49] had their participants perform a DVJ, Pletcher et al. [44] had their participants perform a SL vertical stop jump. Previous findings have reported lower hip and knee flexion angles at IC during SL landing tasks compared with double-leg landing tasks [58–60]. Additionally, SL landing tasks are biomechanically more challenging for the knee joint in comparison to double-leg landing tasks because of greater lower extremity loading, greater motor control and a smaller base of support [48], which in turn could have resulted in pre-pubertal girls adopting a different landing strategy during these tasks.

Despite the findings being equivocal across various studies, landing with less knee flexion increases the load on the ACL because of the increased quadriceps and reduced hamstring muscle activity [61]. An increased knee flexion helps in decreasing the vGRF and rate of force development during jump-landing tasks [62–65], ultimately reducing anterior tibial translation load at the knee. Previous studies have indicated that female athletes tend to land with less knee flexion after 12 years of age during SL stride jump and double-leg stop jump tasks [66, 67]. A prospective study by Hewett et al. [25] reported that female athletes sustaining ACL injuries demonstrated 10.4° less peak knee flexion prior to injury compared with uninjured teammates during a DVJ test. Interestingly, we found moderate evidence for post-pubertal female individuals having higher knee flexion moments compared with pre-pubertal girls, with the latter group displaying less knee flexion at landing in two studies. This highlights that female athletes tend to use a more quadriceps dominant strategy while performing jump-landing tasks as they progress through the maturity stages. Leppanen et al. [17] reported that female athletes with higher peak knee external moment were at an increased risk of injury because of higher quadricep forces. The same authors had previously reported that female athletes also presented with less peak knee flexion [68]. Landing with less knee flexion can result in an increased anterior tibial shear load, especially in early deceleration phases of movement, in turn increasing the possibility of athletes sustaining an ACL injury. Female individuals gain approximately 8–10 kg per year 6–9 months after the onset of the growth spurt [69]. However, this gain in mass is primarily absolute and relative fat instead of lean mass [70]. Therefore, the absence of sufficient strength in addition to the rapid increase in size and weight at about, or near, puberty in female athletes might increase their risk of sustaining ACL injuries [71]. Overall, our review highlights that the knee flexion angle varies at different stages of maturity in female athletes, but the direction of these changes with advancing maturation were not consistent. This highlights the need for further research to better understand the influence of this particular kinematic variable.

### Vertical Ground Reaction Force

We found strong evidence for higher relative peak vGRF in pre-pubertal girls than post-pubertal female individuals. Normalisation of vGRF is performed to account for the differences in various body characteristics such as stature and body mass. Such an approach allows for a valid analysis and comparison of results between different groups/participants [72]. The higher relative vGRF in pre-pubertal athletes could be a result of the normalisation process as post-pubertal female individuals tend to have a higher body mass than pre-pubertal girls. However, this speculation could not be confirmed as the included studies did not provide absolute vGRF values across different maturity groups. The study by Pedley et al. [43] stated that absolute vGRF was higher in more mature groups during a drop jump test, although the absolute values were not reported. Larger force peaks during the early phase of jump-landing tasks are a concern as the majority of ACL injuries have been reported to occur during the first 40 ms of ground contact [73]. An increase in absolute peak vGRF of 100 N has been found to increase the probability of ACL injury by 26% in young female athletes [68]. Therefore, future studies should consider reporting absolute and relative vGRF values across various maturity groups. Further, landing is a motor skill that children do not develop until the age of 12 years and continue to refine as they progress through various developmental stages into adulthood [74]. Previous findings have suggested that the ability to modulate vGRF upon impact and throughout the landing phase improves with the process of ageing owing to contributions from factors such as physical maturity, skill development and experience in performing jumping tasks [75]. Given that the average age of pre-pubertal athletes assessed in the included studies was 10.4 years, the higher relative vGRF in this group could be attributed to their lack of experience in performing the jump-landing tasks as they are still in the process of motor skill acquisition. Koga et al. [73] previously found that elite female athletes who sustained an ACL injury during competitive matches had a peak vGRF of 3.2–4.5 times BW occurring at 40 ms after IC while performing jump-landing tasks. Interestingly, the relative vGRF values were found to range between 3.21 and 8.42 times BW even in pre-pubertal athletes in our review. Therefore, jump-landing techniques with high vGRF, combined with the anatomical, hormonal, biomechanical and neuromuscular changes occurring during the process of growth and maturation, might partly explain the increased incidence of ACL injuries in female adolescent athletes. This further highlights the need for early training interventions in female athletes. As an example, neuromuscular training interventions combining strength and plyometric exercises have been found to reduce the ground reaction forces in female netball players aged 11–13 years (g > − 1.30) [76]. Further, a study by Hewett et al. [77] reported that female adolescents performing plyometric exercises significantly decreased their peak landing forces during a vertical jump by 1.2 times their BW. Participation in such training programmes could help female athletes develop the requisite strength levels and enhanced landing technique as they transition through various stages of maturity, which in turn could reduce the ACL injury rates in this population.

### Other Kinematic/Kinetic Findings

Several biomechanical variables related to the hip and ankle were reported in three studies [24, 39, 48]. Pre-pubertal athletes were found to have a higher hip extension angle at IC compared with post-pubertal female athletes, albeit the differences were non-significant. Although Swartz et al. [48] did not perform an exclusive statistical comparison between pre-pubertal and post-pubertal female athletes, the former were found to have a lower hip flexion angle. However, attenuation of impact forces during landing depends more on active hip flexion than the angle at IC [13]. Given that pre-pubertal girls had higher hip flexion displacement compared with post-pubertal female individuals, the higher relative vGRF could be attributed to the jump-landing technique adopted by pre-pubertal girls. Interestingly, peak hip flexion moment was found to be higher in post-pubertal female individuals, a result that is in line with previous findings in which female athletes between 12 and 21 years of age were found to have a higher peak hip flexion moment compared with their uninjured cohort [17]. However, there was no statistically significant association between this kinetic variable and ACL injury.

With regard to ankle biomechanics, pubertal female athletes did not differ in peak ankle dorsiflexion angle or moment compared to post-pubertal female athletes. A greater ankle plantar flexion angle at IC has been found to reduce the risk of ACL injuries [78, 79]. For instance, trends of ACL injured athletes having a marginally lower ankle flexion compared with uninjured athletes during a DVJ test has been observed in floorball (a form of indoor hockey with five players and a goalkeeper) and basketball athletes between the age of 12 and 21 years [17]. Limited ankle plantar flexion at IC during landing could lead to higher vGRF being subsequently transferred and in turn, loading the knee to a greater extent [78]. However, these interpretations are speculative and the association and variation in magnitude of effect of the above-mentioned variables on knee biomechanics and ACL injury risk across different stages of maturity are still unknown.

### Limitations

While the current review has provided novel analyses of the existing data, certain limitations need to be acknowledged. First, the maturity level of the athletes was classified using several different scales. Even when studies used the same method [25, 49], there were variations in the manner of interpreting the maturity outcome. However, we reduced the heterogeneity of the findings by limiting our review to studies that had clearly reported the scale used for measuring the maturity status of the participants. As an example, a study by Hass et al. [66] in which pubertal and post-pubertal female individuals had been compared was excluded from the review because they did not provide information regarding the scale used for estimating maturity. Second, we could not perform a meta-analysis of the reported kinematic and kinetic variables because of the variation in the study design; the mode of data collection varied across the included studies and certain variables were not reported in all studies. Such a quantitative analysis would help to identify the standardised magnitude of changes of the reported biomechanical variables across various stages of maturity. However, we have aimed to summarise all the kinematic and kinetic variables reported in the literature across various stages of maturity during jump-landing tasks, and in doing so have highlighted the most commonly researched kinematic and kinetic variables reported across various maturity groups in young female athletes.

### Future Research Directions

Jump-landing tasks are usually conducted as part of screening tests to identify athletes at a higher risk of sustaining injuries [80]. Several tests such as a vertical jump, DVJ and tuck jump are routinely performed as part of the screening process. For young and inexperienced athletes, it should be noted that more demanding jumping tasks (e.g. SL jumps) might be difficult to perform without the requisite levels of motor competence. Therefore, future studies should also consider the role of training and sporting experience of the athlete prior to performing various SL and double-leg jump-landing tasks as part of the screening process. Such an approach should provide further clarity on the suitability of a particular jumping test for athletes at certain stages of maturity/technical competence.

Numerous SL and double-leg landing tasks were performed across various studies included in our review. Task constraints play a crucial role in influencing the biomechanical demands during these tasks [81]. The variation in the testing protocols implemented in the studies could be one of the reasons for the strength of evidence being conflicting for a few kinematic and kinetic variables in our review. However, when the same jump-landing task was performed across various studies, there was an improvement in the level of strength of evidence. As an example, we found strong evidence for higher peak knee abduction angle in post-pubertal female individuals as participants in five of the six studies [21, 25, 38, 40, 49] that reported this variable had performed the same DVJ test. Therefore, further research that uses common jump-landing protocols is required to better understand the associations between the biomechanical variables reported in our study and ACL injury.

We found only three studies that had compared the changes in hip and ankle biomechanics across various stages of maturity. The hip and ankle joint play a key role in influencing knee motion during the deceleration phase of jump-landing tasks. However, the strength of evidence for the majority of hip and ankle biomechanical variables ranged from limited to very limited. Therefore, future research is needed to better understand how changes in kinetics and kinematics at the hip and ankle may contribute to ACL injury risk at different stages of maturity.

## Conclusions

We found strong evidence for a higher peak knee abduction angle, knee abduction and internal rotation moment in post-pubertal female athletes. There was also strong evidence for pre-pubertal girls having higher peak vGRF compared with the athletes in the more mature groups. These findings highlight that growth and maturation influence landing biomechanics, which may increase injury mechanisms at different stages of development. It might be beneficial to incorporate strength and conditioning training programmes from a young age in order to help reduce the magnitude of alterations in multiplanar landing biomechanics as female athletes progress through various stages of maturity. However, further research is required to achieve a better understanding of the association between hip, knee and ankle biomechanics and ACL injury across different stages of maturity.

### Supplementary Information

Below is the link to the electronic supplementary material.

Supplementary file1 (DOCX 17 KB)

Supplementary file2 (DOCX 25 KB)

## Declarations

### Availability of data and material

All data and material reported in this systematic review are only from peer-reviewed publications. All the extracted data have been provided in Tables 1 and 5 of the ESM.
