# Supplementary material for: Changes in Lower Limb Biomechanics Across Various Stages of Maturation and Implications for ACL Injury Risk in Female Athletes: a Systematic Review
Source: Sports Med. 2024 Apr 26;54(7):1851–76. doi: 10.1007/s40279-024-02022-3 (PMC11257789; doi:10.1007/s40279-024-02022-3)
Supplement: Supplementary file 1 — Supplementary file1 (DOCX 17 KB) [file 40279_2024_2022_MOESM1_ESM.docx]

**Supplementary Material Appendix S1:** Search strategy used for searching the relevant articles across various databases.

**PUBMED (Medline) Search Strategy**

("growth"[Title/Abstract] OR "Matur*"[Title/Abstract] OR "Pubert*"[Title/Abstract] OR "Young"[Title/Abstract] OR “Youth” [Title/Abstract] OR “Adolescen*” [Title/Abstract] OR “Tanner stage*”[Title/Abstract] OR “Sex” [Title/Abstract] OR “Female” [Title/Abstract] OR “Girl*”[Title/Abstract]) AND ("Biomechanic*"[Title/Abstract] OR "Mechanic*"[Title/Abstract] OR "kinematic*"[Title/Abstract] OR "kinetic*"[Title/Abstract]) AND ("ACL"[Title/Abstract] OR "anterior cruciate ligament"[Title/Abstract] OR "Knee"[Title/Abstract] OR "lower extremit*"[Title/Abstract] OR “Hip” [Title/Abstract] OR “Ankle” [Title/Abstract] OR “Leg” [Title/Abstract]) AND (“jump landing”[Title/Abstract] OR “jumping”[Title/Abstract] OR “jumping” [Title/Abstract] OR “land”[Title/Abstract] OR “landing”[Title/Abstract]) AND ("injur*"[Title/Abstract] OR "Inciden*"[Title/Abstract] OR "Risk factor*"[Title/Abstract]) = **554 results**

**SCOPUS Search Strategy**

TITLE-ABS-KEY ("growth" OR "Matur*" OR "Pubert*" OR "Young" OR “Youth” OR “Adolescence” OR “Tanner stage*” OR “Sex” OR “Female” OR “Girl”) AND TITLE-ABS-KEY("Biomechanic*" OR "Mechanic*" OR "kinematic*” OR "kinetic*") AND TITLE-ABS-KEY ("ACL" OR "anterior cruciate ligament" OR "Knee" OR "lower extremit*” OR “Hip” OR “Ankle” OR “Leg”) AND TITLE-ABS-KEY (“jump landing” OR “jumping” OR “jumping” OR “land” OR “landing”) AND TITLE-ABS-KEY (“injur*" OR "Inciden*" OR "Risk factor*") = **1,658 results**

**SPORTDiscus Search Strategy**

("growth" OR "Matur*" OR "Pubert*" OR "Young" OR “Youth” OR “Adolescence” OR “Tanner stage*” OR “Sex” OR “Female” OR “Girl*”) AND ("Biomechanic*" OR "Mechanic*" OR "kinematic*” OR "kinetic*") AND ("ACL" OR "anterior cruciate ligament" OR "Knee" OR "lower extremit*” OR “Hip” OR “Ankle” OR “Leg”) AND (“jump landing” OR “jumping” OR “jumping” OR “land” OR “landing”) AND (“injur*" OR "Inciden*" OR "Risk factor*") = **718 results**

**EMBASE Search Strategy**

(Growth OR Matur* OR Pubert* OR Young OR Youth OR Adolescen* OR Tanner stage* OR Sex OR Female OR Girl*) AND (Biomechanic* OR Mechanic* OR kinematic* OR kinetic*) AND (ACL OR anterior cruciate ligament OR Knee OR lower extremit* OR Hip OR Ankle OR Leg) AND (jump landing OR jumping OR jumping OR land OR landing) AND (injur* OR Inciden* OR Risk factor*) = **1,470 results**

**CINAHL Search Strategy**

("growth" OR "Matur*" OR "Pubert*" OR "Young" OR “Youth” OR “Adolescence” OR “Tanner stage*” OR “Sex” OR “Female” OR “Girl*”) AND ("Biomechanic*" OR "Mechanic*" OR "kinematic*” OR "kinetic*") AND ("ACL" OR "anterior cruciate ligament" OR "Knee" OR "lower extremit*” OR “Hip” OR “Ankle” OR “Leg”) AND (“jump landing” OR “jumping” OR “jumping” OR “land” OR “landing”) AND (“injur*" OR "Inciden*" OR "Risk factor*") = **917 results**

**Web of Science Search Strategy**

TITLE-ABS-KEY("growth" OR "Matur*" OR "Pubert*" OR "Young" OR “Youth” OR “Adolescence” OR “Tanner stage*” OR “Sex” OR “Female” OR “Girl”) AND TITLE-ABS-KEY ("Biomechanic*" OR "Mechanic*" OR "kinematic*” OR "kinetic*") AND TITLE-ABS-KEY ("ACL" OR "anterior cruciate ligament" OR "Knee" OR "lower extremit*” OR “Hip” OR “Ankle” OR “Leg”) AND TITLE-ABS-KEY (“jump landing” OR “jumping” OR “jumping” OR “land” OR “landing”) AND TITLE-ABS-KEY(“injur*" OR "Inciden*" OR "Risk factor*") = **973 results**
