# Supplementary material for: Changes in Lower Limb Biomechanics Across Various Stages of Maturation and Implications for ACL Injury Risk in Female Athletes: a Systematic Review
Source: Sports Med. 2024 Apr 26;54(7):1851–76. doi: 10.1007/s40279-024-02022-3 (PMC11257789; doi:10.1007/s40279-024-02022-3)
Supplement: Supplementary file 2 — Supplementary file2 (DOCX 25 KB) [file 40279_2024_2022_MOESM2_ESM.docx]

| **Supplementary Material Table S1:** Downs and Black checklist quality assessment criteria | |
| --- | --- |
| **Question** | **Rating criteria** |
| **Reporting** | |
| 1. Is the hypothesis/aim/objective of the study clearly described? | 1- The aim of the study is clearly described in the Introduction section  0- No information is provided |
| 2. Are the main outcomes to be measured clearly described in the Introduction or Methods sections? | 1- If the outcomes to be measured are mentioned in the Methods section.  0- If this information is mentioned in the Results section first. |
| 3. Are the characteristics of the subjects included in the study clearly described? | 1- If the inclusion/exclusion criteria for the study is provided  0- If information such as age, height and weight are provided but inclusion/exclusion criteria not mentioned. |
| 5. Are the distributions of principle confounders in each group of subjects to be compared clearly described? | 1- Maturity level is provided  0- No information is provided |
| 6. Are the main findings of the study clearly described? | 1- If the outcome data was clearly defined  0- If there were discrepancies in determining the outcome |
| 7. Does the study provide estimates of the random variability in the data for the main outcome? | 1- If studies have provided Standard deviation, 95%CI or Standard error values  0- If no such information is provided |
| 10. Have actual probability values been reported (e.g. 0.035 rather than < 0.05) for the main outcomes except where the probability is less than 0.001? | 1- If exact p-value for a particular outcome is provided.  0- If this information is not available. |
| **External validity** | |
| 11. Were the subjects asked to participate in the study representative to the entire population from which they were recruited? | 1- If the population was females with no restriction on the sport they participated (2 or more)  0- If the population was females from on specific sport |
| 12. Were those subjects who were prepared to participate representative of the entire population from which they were recruited? | 1- If the number of participants who refused was stated in the study  0- No information is provided |
| **Internal validity** | |
| 15- Was an attempt made to blind those measuring the main outcome? | 1- If an attempt was made to blind the people conducting the experiment  0- No information was provided |
| 16- If any of the results was based on “data dredging”, was this made clear? | 1- Any analyses that had not been planned at the outset of the study should be clearly indicated  0- No |
| 18. Were the statistical tests used to assess the main outcomes appropriate? | 1- The statistics used in the study were appropriate for analysing the data set  0- No |
| 20. Were the main outcome measures used accurate (valid and reliable)? | 2- Accuracy and methods are reported  1- Only Methods are reported  0- No such information |
| **Internal validity- Confounding (risk of bias)** | |
| 21. Were the subjects (e.g. the two groups to be compared) recruited from the same population? | 1- If they were from the same clinic or school etc.  0- If they were from different locations or no information was provided |
| 22. Were the study subjects (the two groups to be compared) recruited over the same period of time? | 1- If this information was provided in the study  0- Studies that does not specify the time period over which patients were recruited |
| 25. Were there adequate adjustments for confounding in the analyses from which the main findings were drawn? | 1- If the effect of the main confounders was investigated or confounding was demonstrated  0- If the effect of the main confounders was not investigated or confounding was demonstrated but no adjustment was made in the final analysis. |
| **Power analysis** | |
| 27. Did the study have sufficient power to detect a clinically important effect? | 1- Performed power or sample size analysis  0- No information is provided |
